# Crystallographic and computational investigation of a bent-core Schiff base Ni(ii) complex with DNA and protein binding studies

**DOI:** 10.1039/d5ra07894f

**Published:** 2026-03-05

**Authors:** Kamrun Nahar Alia, Bugra Koknarugmani Debbarma, Sourav Nath, Subhadip Roy, Alan R. Kennedy, Suman Adhikari, Malavika S. Kumar, Avijit Kumar Das, Samiyara Begum, Golam Mohiuddin

**Affiliations:** a Department of Chemistry, University of Science & Technology Meghalaya Ri-Bhoi Meghalaya 793101 India golammohiuddin.ustm@gmail.com samiyara.ustm@gmail.com; b Department of Chemistry and Vivekananda Centre for Research, Ramakrishna Mission Residential College Narendrapur Kolkata-700103 India; c Department of Chemistry, Govt. Degree College Dharmanagar Tripura(N)-799253 India sumanadhi@gmail.com; d Department of Chemistry, The ICFAI University Tripura Kamalghat, Mohanpur Agartala 799210 Tripura India; e Department of Pure and Applied Chemistry, University of Strathclyde 295 Cathedral Street Glasgow G1 1XL Scotland UK; f Department of Chemistry, Christ University Hosur Road Bangalore Karnataka 560029 India avijitkumar.das@christuniversity.in

## Abstract

The rational design and synthesis of a three-ring bent-core Schiff base ligand, (*E*)-4-(trifluoromethyl)phenyl-3-((4-butoxy-2-hydroxybenzylidene)amino)-2-methylbenzoate (HL), and its mononuclear Ni(ii) complex, [Ni(L)_2_] (1), are described. The presence of a polar –CF_3_ group and a flexible butoxy chain imparts amphiphilic character to HL and induces aggregation-induced emission (AIE) behavior. Coordination with NiCl_2_ yields a square-planar complex, as confirmed by spectroscopic methods, single-crystal X-ray diffraction analysis, and topological analysis. Fluorescence and SEM studies substantiate the aggregation propensity of HL. Density functional theory (DFT) and natural bond orbital (NBO) analyses reveal pronounced ligand-to-metal charge transfer in (1) and a moderate HOMO–LUMO gap of 4.00 eV, indicative of kinetic stability and optoelectronic relevance. Complex (1) exhibits strong binding affinity toward duplex DNA and serum proteins (BSA and HSA), evidenced by red-shifted fluorescence enhancement at 475 nm and low detection limits (0.075–0.188 µM). Molecular docking further supports stable BSA binding (−8.52 kcal mol^−1^), highlighting the potential of this Ni(ii) system for biomolecular recognition.

## Abstract 2

The rational design and synthesis of a three-ring bent-core Schiff base ligand, (*E*)-4-(trifluoromethyl)phenyl-3-((4-butoxy-2-hydroxybenzylidene)amino)-2-methylbenzoate (HL), and its mononuclear Ni(ii) complex, [Ni(L)_2_] (1), are described.

## Graphical abstract

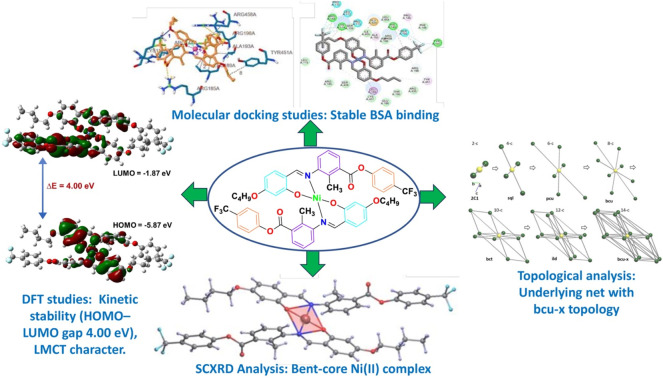

## 1. Introduction

Recent decades have witnessed remarkable advances in coordination chemistry, driven primarily by the strategic design of innovative ligands.^1,2^ Even subtle modifications to ligand electronic or steric properties, such as backbone structure, donor atoms, or bridging groups, can profoundly influence the reactivity and function of metal complexes.^3,4^ Careful selection of ligand and metal is essential for developing inorganic materials with potent pharmacological properties.^5,6^ Bent-shaped (bent-core) molecules typically consist of a central aromatic unit—most often a 1,3-disubstituted phenylene ring—that links two rigid segments at an angle, producing a V-shaped molecular geometry. These compounds are primarily studied as liquid crystalline materials due to their characteristic non-linear, banana-like architecture.^7–9^ The inherent molecular bend gives rise to complex mesomorphic behavior, frequently leading to the formation of B-series mesophases (B1–B8).^10–16^ While extended bent-core systems containing five or more aromatic rings have been widely explored, three-ring bent-core molecules remain comparatively underrepresented in coordination chemistry. Their shorter molecular length and lower aspect ratio introduce synthetic difficulties and can significantly influence mesophase stability. Nevertheless, our previous work demonstrated that polar three-ring bent-core compounds can be successfully synthesized and exhibit both nematic and smectic phases, underscoring their promise for fundamental investigations and functional applications.^17,18^ Moreover, the bent geometries of ligands can create metal-organic coordination patterns that are difficult for linear ligands to accomplish.^19,20^ Interestingly, novel metal-organic frameworks were produced by this fine tuning of ligand-directed strategy, particularly when the bent geometries of ligands contain a well-isolated cavity.^21–23^ In coordination chemistry, Schiff bases are considered privileged ligands because of their many pharmacological properties and intrinsic synthesis advantage.^24–26^ The behavior observed in these three-ring bent core systems seems promising, but deeper investigation into their structure-property relationships with respect to their coordination chemistry is needed for the development of metallodrugs.

The potential of metal-based complexes as drugs has been considerably studied due to their diverse spectral, chemical, and electronic characteristics.^27,28^ HSA and BSA, two important human proteins, function as plasma transporters, and the interaction between plasma proteins and metal-based complexes is important for pharmacology research and drug design.^29^ Furthermore, these proteins' toxicity can be reduced, and their solubility can be increased by binding with metal complexes.^30^ Moreover, the potency of novel metal-based therapeutics depends on the interaction between DNA and metal-based complexes.^31^ Many of the anticancer drugs currently in use target DNA reversibly, and the ability to bind DNA is crucial while looking for novel anticancer drugs.^32,33^ Metal complexes primarily interact with DNA *via* non-covalent contacts, such as surface/electrostatic interactions, intercalation of planar aromatic ring systems between base pairs, and groove binding contacts, which entail direct contacts between the bound molecule and the edges of the base pair in either the minor (A–T) or major (G–C) grooves.^34,35^ Metal-based complexes with aromatic side moieties have received special interest because they can attach to DNA through both metal ion coordination and aromatic moiety intercalation.^36^ Furthermore, drug recognition with proteins becomes essential since it provides important evidence to control the therapeutic efficiency of medications.^37^ Exploring the interaction of metal-based compounds with serum albumin is particularly crucial since it provides an extra benefit for using them as a possible medication, because it is well acknowledged that the plasma protein HSA functions as an efficient drug carrier.^38,39^ Nickel has been recognized as one of the most important micronutrients for plant growth and development, and its absence cannot be replaced by any other nutrient.^40^ Recently, after the discovery of several nickel-containing or nickel-dependent enzymes, the coordination chemistry of nickel complexes has greatly advanced.^41,42^ Ni(ii) complexes have shown potential anticancer, antibacterial, antimicrobial, and antifungal properties, and have drawn more attention from researchers studying bio-inorganic chemistry.^43–45^ By interacting with proteins or DNA, Ni(ii) complexes exert their anticancer effects.^46^

Herein, we report the design, synthesis, and characterization of a new three-ring-based bent-core Schiff base ligand, (*E*)-4-(trifluoromethyl)phenyl 3-((4-butoxy-2-hydroxybenzylidene)amino)-2-methylbenzoate (HL) and its mononuclear Ni(ii) complex, [Ni(L)_2_] (1). The new three-ring based bent-core Schiff base ligand was thoroughly characterised by spectroscopic methods (FT-IR, ^1^H NMR, ^13^C NMR, and elemental analysis) while its corresponding Ni(ii) complex (1) was explicated by using spectroscopic methods (FT-IR, ESI-MS, and elemental analysis), single-crystal X-ray diffraction analysis, and topological analysis. The aggregation behaviour of the bent-core Schiff base ligand HL was studied by fluorescence methods and SEM analysis. The electronic properties of 1 have been demonstrated by theoretical investigations based on DFT calculations. Moreover, the binding ability of the Ni(ii) complex (1) with DNA and proteins was also investigated. The potential binding affinity of 1 with the target protein receptor (4JK4) was further examined using a molecular docking method. This work advances the understanding of bent-core ligands in metal complex design and their potential as biomolecular recognition scaffolds.

## 2. Experimental sections

### 2.1 Design and synthesis of bent-core Schiff base ligand (HL) and its Ni(ii) complex (1)

**Scheme 1:**
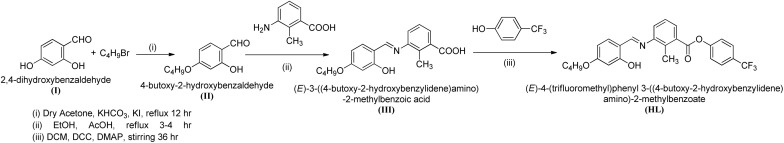
Synthesis of the bent-core Schiff base ligand (HL).

We have designed a novel bent-core Schiff base ligand (HL) and its corresponding Ni(ii) complex [Ni(L)_2_] (1) involving a minimum number of three phenyl rings. In order to accomplish a perfect bend within the molecular architecture, the three phenyl rings in the molecular design also help to distribute aromatic rings symmetrically with regard to the central core ring. The incorporation of a methyl (–CH_3_) group at the central core's kink point results in an extended bent angle of 145°.^17,18^ The three-ring based bent-core Schiff base ligand (HL) has been synthesised following the reaction Scheme 1.

### 2.2 Synthesis of 4-butoxy-2-hydroxybenzaldehyde^47^(II)

2,4-Dihydroxybenzaldehyde (1.38 g, 10 mmol), 1-bromobutane (1.078 mL, 10 mmol), KHCO_3_ (2.00 g, 20 mmol), and a catalytic amount of KI were added in dry acetone (50 mL), and the reaction mixture was refluxed for 24 h to give the product 4-butoxy-2-hydroxybenzaldehyde. The mixture was filtered to remove the insoluble solid and was further purified *via* column chromatography using silica gel (60–120 mesh) with petroleum ether as the solvent. The product was obtained as a pale-yellow liquid. Yield: 1.29 g, 66.6%.

### 2.3 Synthesis of the Schiff base (III)

The intermediate Schiff base [(*E*)-3-((4-butoxy-2-hydroxybenzylidene)amino)-2-methylbenzoic acid] was synthesized by refluxing a mixture of an ethanolic solution of 4-butoxy-2-hydroxybenzaldehyde (1.94 g, 10 mmol) and 3-amino-2-methylbenzoic acid (1.51 g, 10 mmol) with a few drops of glacial CH_3_COOH.^48^ The yellow precipitate was then recrystallised in ethanol. Yellow solid, yield: 1.98 g, 60.5%.

### 2.4 Synthesis of the bent-core Schiff base ligand (HL)

The Schiff base [(*E*)-3-((4-butoxy-2-hydroxybenzylidene)amino)-2-methylbenzoic acid] (1.64 g, 5 mmol), 4-(trifluoromethyl)phenol (0.81 g, 5 mmol), and 4-dimethylaminopyridine (DMAP) were all taken in 1 eq. and was dissolved in dry DCM and stirred for about 30 min at 0 °C. Then *N*,*N*′-dicyclohexylcarbodiimide (DCC) (1.03 g, 5 mmol) was added and kept for 30 hours with stirring in an inert condition at r.t to yield the product. Then the residue was filtered off, and the solvent was removed to obtain the solid yellow precipitate. Later, the precipitate was recrystallized using ethanol to get the pure product. *R*_f_ = 0.508 cm.

### 2.5 Bent-core Schiff base ligand (HL)

Yield: 1.67 g, 71%. M. P = 98 °C. Anal. calc. For C_26_H_24_F_3_NO_4_: C, 66.24; H, 5.13; N, 2.97. Found: C, 66.22; H, 5.17; N, 3.03. FT-IR (*ν* cm^−1^, KBr): 3460 [*ν*_phenoloic(O–H)_], 1743 [*ν*_(C=O)_], 1624 (imine *ν*_(C=N)_ stretching), 1331 [*ν*_(C–O)_]. ^1^H NMR (400 MHz, CDCl_3_, *δ* in ppm): 13.46 (s, 1H, –OH), 8.44 (s, 1H, –CH=N–), 7.97 (d, 1H, Ar–H), 7.72 (d, 2H, Ar–H), 7.36–7.39 (m, 3H, Ar–H), 7.26–7.31 (m, 2H, Ar–H), 6.51 (t, 2H, Ar–H), 4.02 (t, 2H, –OCH_2_), 2.66 (s, 3H, –CH_3_), 1.78 (q, 2H, –CH_2_^−^), 1.50 (m, 2H, –CH_2_^−^), 0.98 (t, 3H, –CH_3_). ^13^C NMR (100 MHz, CDCl_3_, *δ* in ppm): 165.4, 163.9, 163.5, 162.7, 153.3, 149.6, 134.5, 133.6, 128.4, 126.8, 126.6, 124.7, 122.9, 122, 112.9, 107.7, 101.5, 68.0, 31.1, 19.2, 13.8.

### 2.6 Synthesis of the Ni(ii) complex (1)

**Scheme 2:**
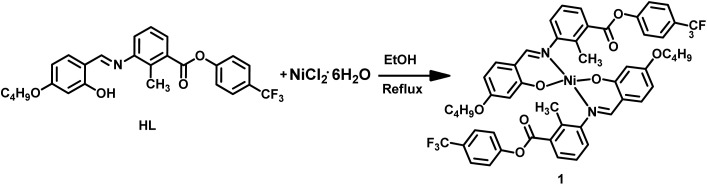
Synthesis of the Ni(ii) complex (1).

In a round-bottom flask, ligand (HL) (0.06 g, 0.000127 mmol) was dissolved in ethanol (10 mL). To this solution, nickel(ii) chloride hexahydrate (NiCl_2_·6H_2_O) (0.015 g, 0.0000635 mmol), dissolved in ethanol (10 mL), was added dropwise (Scheme 2). The resulting mixture was refluxed for 4 hours. The volume of the reaction mixture was reduced, and the resulting greenish-yellow precipitate was collected by filtration, washed with ethanol (5 mL), and air-dried. Next, the precipitate was dissolved in dimethyl formamide (DMF), and after 3 days, single crystals of complex 1 were obtained. The reaction was also examined using Ni(OAc)_2_·4H_2_O as an alternative metal precursor under identical experimental conditions. The resulting product exhibited identical FT-IR spectroscopic features to those obtained using NiCl_2_·6H_2_O, indicating the formation of the same coordination species. However, single crystals of sufficient quality for X-ray diffraction analysis were obtained only when NiCl_2_·6H_2_O was employed.

### 2.7 Complex 1

Yield: 80%. Anal*.* calc. For C_52_H_46_F_6_N_2_NiO_8_: C, 62.48; H, 4.64; N, 2.80. Found: C, 62.45; H, 4.68; N, 2.77. FT-IR (*ν* cm^−1^, KBr): 1751 [*ν*_(C=O)_], 1629 (imine *ν*_(C=N)_ stretching), 1338 [*ν*_(C–O)_], 637 [*ν*_(Ni–N)_]. HRMS: (*m*/*z*): 998.2759 (M^+^).

## 3. Results and discussions

### 3.1 Spectral characterization of the bent-core Schiff base ligand (HL) and its Ni(ii) complex, [Ni(L)_2_] (1)

The synthesized bent-core ligand (HL) has been well characterized by ^1^H NMR spectroscopic analysis, as shown in Fig. S1. The presence of a singlet peak at *δ* = 13.46 ppm downfield region confirms the presence of an H-bonded –OH proton, a singlet peak at *δ* = 8.44 ppm signifies the presence of an imine proton (–CH=N–), and another singlet peak at *δ* = 2.66 ppm confirms the –CH_3_ protons at the kink/bay position of the central phenyl ring. The triplet peak at *δ* = 4.02 ppm confirms the presence of –OCH_2_^−^ protons of the alkoxy chain at one end. ^13^C NMR spectroscopic data (Fig. S2) also substantiate the formation of ligand HL. In FT-IR spectroscopy (Fig. S3) band at 1624 cm^−1^ corresponds to imine (–CH=N–) stretching frequency, whereas the band at 1743 cm^−1^ is ascribed to ester (–C=O) stretching frequency. In the FT-IR spectrum of complex 1, the imine (–CH=N–) stretching frequency shifted to 1629 cm^−1^, signifying the binding of the imine nitrogen to the nickel atom (Fig. S4).^49,50^

UV-vis absorption studies were performed for the free ligand (HL) and the corresponding Ni(ii) complex. The free ligand exhibits two characteristic absorption bands at 296 nm and 371 nm; however, upon complexation with Ni(ii), a gradual decrease in absorbance is observed at both wavelengths. The observed decrease in absorbance upon coordination of Ni^2+^ with the Schiff base ligand can primarily be attributed to the involvement of the imine nitrogen lone pair and hydroxy group in metal binding, which reduces the intensity of ligand-centered π → π* and *n* → π* transitions, leading to a hypochromic effect. In addition, metal-ligand coordination perturbs the ligand conjugation and rigidity and introduces weak, Laporte-forbidden d–d transitions characteristic of Ni(ii), which are significantly less intense than the transitions of the free ligand, resulting in an overall reduction in absorbance intensity.^51^

### 3.2 Aggregation and solvatochromic studies of bent-core Schiff base ligand (HL)

**Fig. 1:**
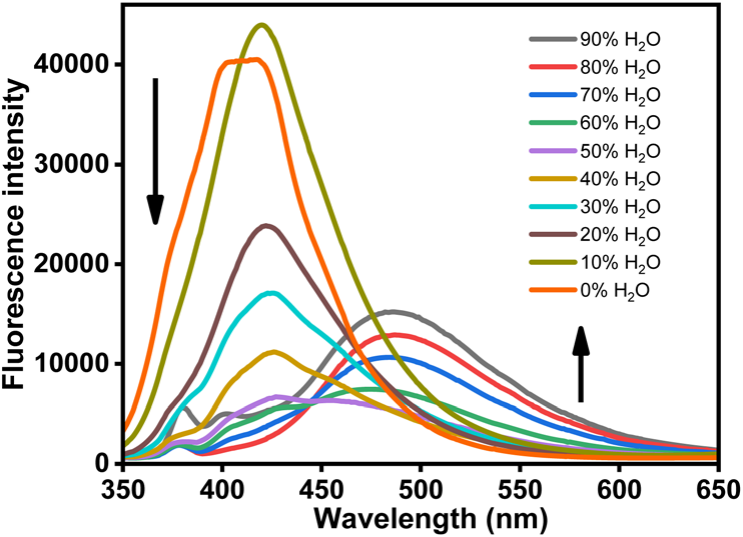
Fluorescence spectra of ligand HL (*c* = 2.0 × 10^−5^ M) in CH_3_CN–H_2_O mixtures with different H_2_O volume fractions (*λ*_ex_ = 340 nm).

A remarkable change in fluorescence was observed for ligand (HL) as it states changes from monomolecular to aggregation state (Fig. 1).^52^ The study was carried out in CH_3_CN–H_2_O mixtures with an increase in water concentration. The fluorescence intensity at 420 nm was found to decrease with an increase in water concentration until the water fraction was till 50% followed by the appearance of a red-shifted emission peak at 486 nm (Δ*λ* = 66 nm) from 60% water fraction by the emission enhancement with increase in water fraction till 90%. The initial fluorescence intensity in CH_3_CN medium is due to intramolecular C=N isomerization. Upon successive increase in H_2_O content with high polarity, there is a significant red shift of the emission band owing to inhibition of the C=N isomerization and restriction of the Intramolecular Rotation (RIR) mechanism.^53^

**Fig. 2:**
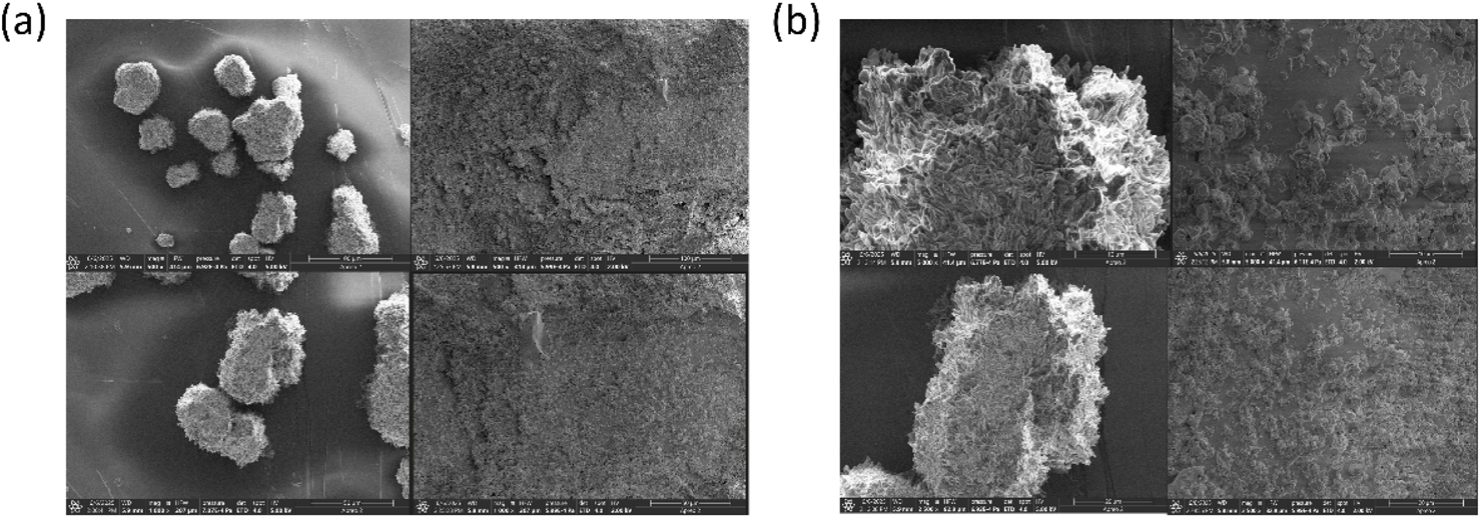
SEM images of ligand HL (*c* = 2 × 10^−5^ M) prepared from its solution in (a) 100% CH_3_CN and (b) 90% H_2_O–CH_3_CN mixture.

Moreover, the SEM images offer clear insight into the aggregation behavior of the ligand HL in aqueous environments.^54^ In pure CH_3_CN, the images reveal the formation of tightly packed and well-organized aggregates (Fig. 2a). In contrast, when using CH_3_CN–H_2_O mixtures (10 : 90, v/v) with higher water content, larger assemblies of hydrophobic fluorophores are observed (Fig. 2b). These findings also help explain the emergence of two distinct fluorescence emissions corresponding to the different aggregation states of the ligand in varying CH_3_CN–H_2_O ratios.

The UV-vis absorption spectra of HL recorded in different solvents clearly demonstrate pronounced solvatochromic behavior, with both the intensity and spectral profile strongly dependent on solvent polarity. Among the tested solvents, HL shows high absorbance in CHCl_3_ and the lowest absorbance in CH_3_CN, while both CH_3_OH and C_2_H_5_OH exhibit moderate absorbance. This solvent-dependent behavior arises because the weakly polar and non-coordinating nature of CHCl_3_ preserves the planarity and π-conjugation of HL, leading to enhanced π → π* transitions. In contrast, strong dipolar solvation in CH_3_CN stabilizes the ground state more effectively, reducing transition probability and resulting in lower absorbance. The polar protic solvents CH_3_OH and C_2_H_5_OH show intermediate absorbance due to hydrogen-bonding interactions that partially perturb conjugation while simultaneously stabilizing the excited state (Fig. S13 and S14).^55^

### 3.3 Crystal structure description and topological studies of the Ni(ii) complex (1)

**Fig. 3:**
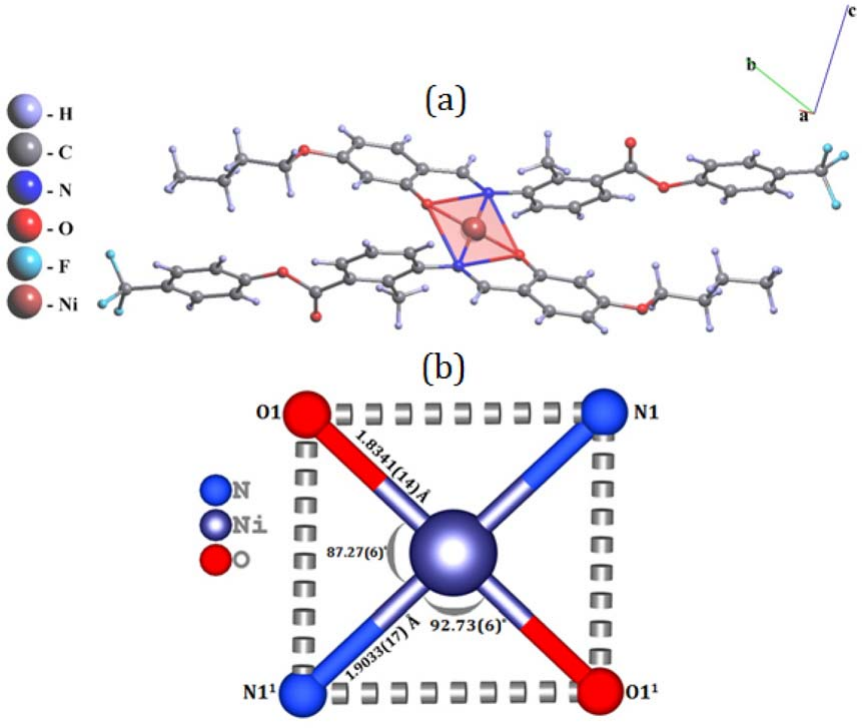
(a) Molecular structure of Ni(ii) complex 1; the ligand contains a CF_3_ substituent, and only the major component is shown. (b) Core view of Ni(ii) complex 1 with bond distance and bond angles. Primed atom labels refer to symmetry-equivalent positions generated by the inversion center.

Crystallography details are represented in Table 1. In complex 1, the Ni(ii) center lies on an inversion center, rendering only half of the molecule crystallographically independent; the remainder is generated through the symmetry operation 1 − *x*, 1 − *y*, 1 − *z*. The asymmetric unit (Fig. S6) comprises a Ni(ii) ion coordinated by the imine nitrogen and phenoxido oxygen atoms of the deprotonated Schiff base ligand (L). The symmetry-related coordination results in a square planar geometry around the Ni(ii) center.^56^ The Ni–O1 and Ni–N1 bond lengths are 1.8341(14) Å and 1.9033(17) Å, respectively. The coordination angles are O1–Ni–N1 = 92.73(6)°, O1–Ni–N1^1^ = 87.27(6)°, and O1–Ni–O1^1^ = N1–Ni–N1^1^ = 180.0°, as detailed in Table S1 and illustrated in Fig. 3.

**Table 1:** Crystal data and structure refinement for Ni(ii) complex, [Ni(L_2_)] (1)

Identification code	1
Empirical formula	NiF_6_O_8_N_2_C_52_H_46_
Formula weight	999.637
Temperature/K	100.15
Crystal system	Triclinic
Space group	*P*1̄
*a*/Å	6.5661(2)
*b*/Å	11.9787(3)
*c*/Å	15.7030(6)
*α*/°	68.636(3)
*β*/°	87.690(3)
*γ*/°	77.073(3)
Volume/Å^3^	1119.92(7)
*Z*	1
*ρ*_calc_ g cm^−3^	1.482
µ/mm^−1^	1.361
*F*(000)	517.0
Crystal size per mm^3^	0.18 × 0.08 × 0.03
Radiation	Cu Kα (*λ* = 1.54184)
2*θ* range for data collection/°	6.04 to 138.06
Index ranges	−7 ≤ *h* ≤ 7, −11 ≤ *k* ≤ 14, −18 ≤ *l* ≤ 18
Reflections collected	9683
Independent reflections	4098 [*R*_int_ = 0.0294, *R*_sigma_ = 0.0396]
Data/restraints/parameters	4098/95/346
Goodness-of-fit on *F*^2^	1.044
Final *R* indexes [I ≥ 2*σ* (I)]	*R*_1_ = 0.0421, w*R*_2_ = 0.1034
Final *R* indexes [all data]	*R*_1_ = 0.0516, w*R*_2_ = 0.1092
Largest diff. peak/hole/e Å^−3^	0.47/−0.56

Considering the well-established redox non-innocent behavior of imine functionalities^57,58^ in transition metal complexes, particularly in Ni(ii) systems, the bonding parameters associated with the azomethine (–CH=N–) fragment in complex 1 were carefully examined. The crystallographically determined C=N bond length in complex 1 is 1.308(3) Å, which lies well within the typical range reported for neutral imine coordination and is significantly shorter than values commonly associated with reduced or radical imine species. Furthermore, the observed C=N bond length is consistent with those reported for structurally related Ni(ii) Schiff base complexes exhibiting innocent ligand behavior, as documented in previous studies.^59,60^ Collectively, these structural features confirm that the Schiff base ligand in complex 1 remains electronically neutral upon coordination, supporting a classical Ni(ii) oxidation state without significant imine-centered redox involvement.

**Fig. 4:**
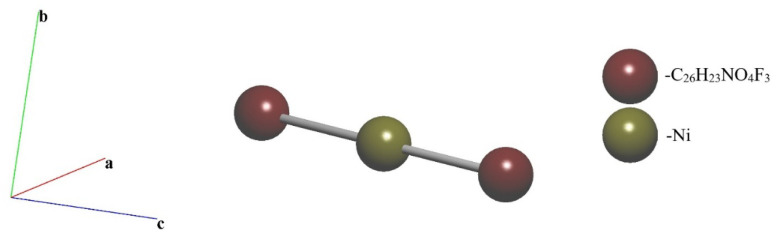
Underlying net of the compound in the standard representation.

Coordination formula of 1 is represented by AB^01^ according to topological analysis. The standard representation of the structure resulted in the 2-nodal net of the 1,2 M3-1 topological type (Fig. 4).

**Fig. 5:**
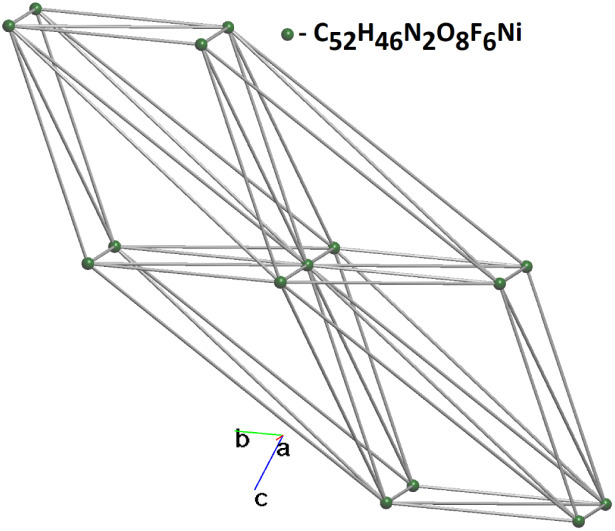
The bcu-x simplified net.

By considering all intermolecular interactions during the simplification procedure, a detailed description of the molecular packing can be obtained. Utilizing the subroutine implemented in ToposPro, various subnets can be derived from the underlying net by retaining only the edges with weights equal to or greater than a specified threshold. The results of the topological analysis revealed that the underlying net corresponds to the bcu-x topological type, with a point symbol of {3^36^.4^44^.5^7^} (Fig. 5).

**Fig. 6:**
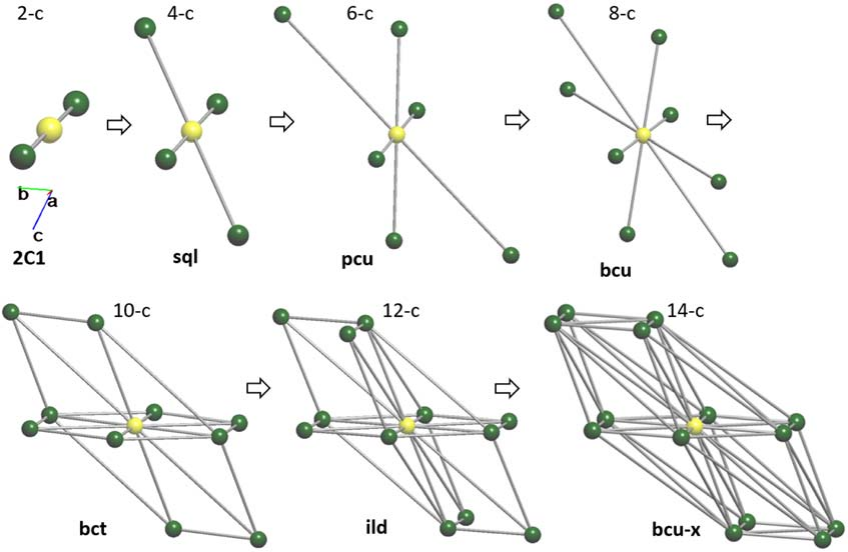
Order of the subnets that describe the packing of the structure on different levels of *Ω*_i_. The central atom is highlighted in yellow.

By applying multilevel analysis,^61^ the resulting sequence of subnets describing the structural packing at different levels of solid angle coverage (*Ω*_i_, %) was obtained, as summarized in Table 2. Fig. 6 illustrates the formation of a 14-c net as a function of the solid angle value, suggesting a possible mechanism for the formation of the final structure.

**Table 2:** Multilevel analysis of molecular complex packing as monomer

No	Node degrees	*Ω*_i_, %	Dimensionality of net	Topological type
1	14-c	2.56	3D	bcu-x
2	12-c	4.94	3D	ild
3	10-c	4.95	3D	bct
4	8-c	5.18	3D	bcu
5	6-c	5.19	3D	pcu
6	4-c	6.19	2D	sql
7	2-c	20.99	1D	2C1

## 4. Theoretical studies

### 4.1 Molecular electrostatic potential (MEP) and structural optimization

**Fig. 7:**
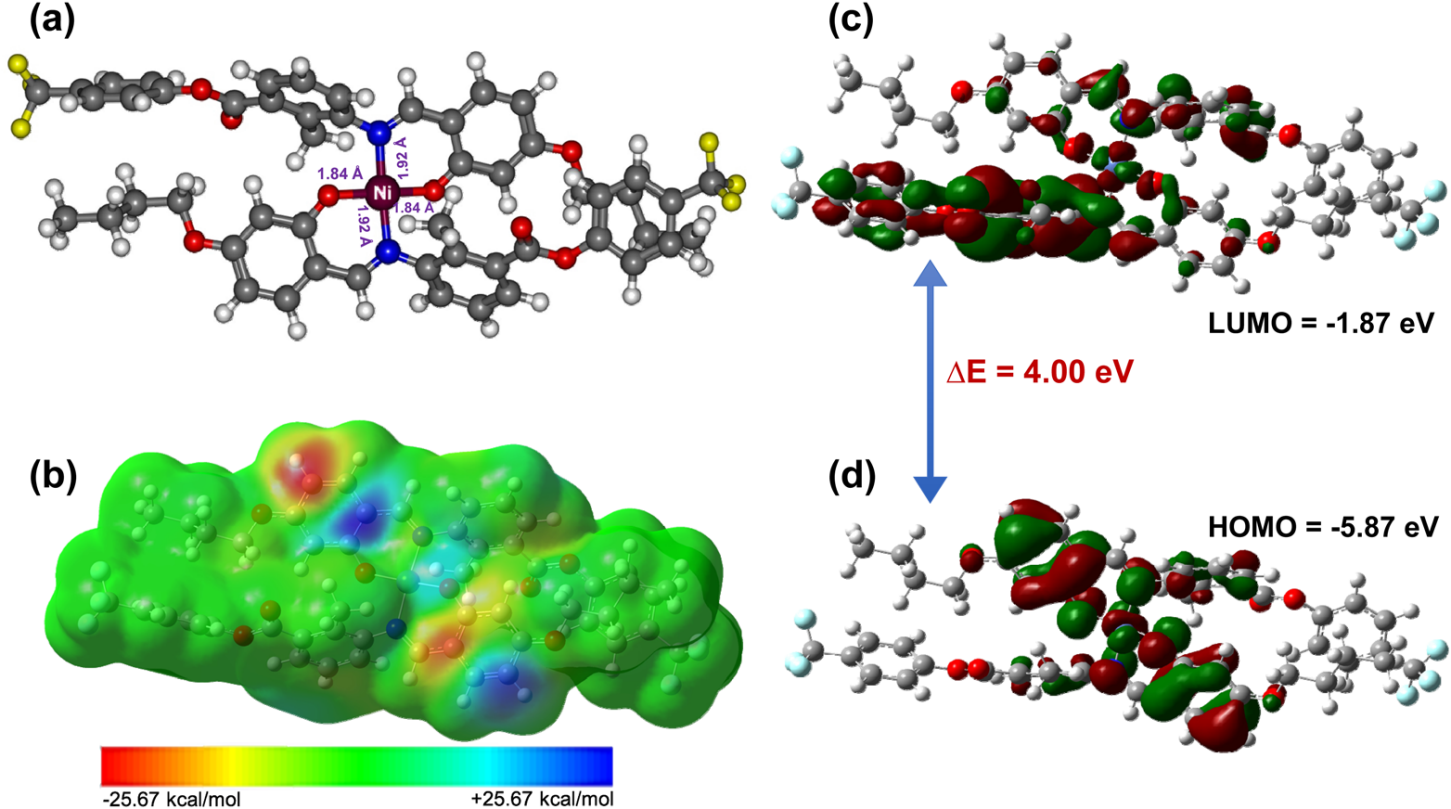
DFT study exhibiting (a) optimized geometry of 1, (b) MEP surface, (c) LUMO orbital and, (d) HOMO orbital with energy gap between LUMO–HOMO.

To investigate different important electronic parameters of [Ni(L)_2_] (1), the DFT calculation was performed in the gas phase using Gaussian 09 software,^62^ where initial coordinates for DFT optimization were taken from the single crystal X-ray coordinates. The Cartesian coordinates of 1 optimized at M06/def2svp^63,64^ is presented in Table S3. The calculated vibrational frequency at the same level of theory shows that the complex exhibits no imaginary frequency. The energy optimized structure of 1 is shown in Fig. 7. It has been found that in the gas-phase optimized geometry of 1, is formed with square planar geometry having both the Ni–N (blue color) bond distance 1.92 Å and both Ni–O (red color) bond distance 1.84 Å. Fig. 7 shows the MEP surface of 1 (GaussView 5.0). The positive electrostatic potential is homogenously distributed in the majority of all over the molecular surfaces, except for a few prominent negative centers (with red colour contours).^65^

Subsequent geometry optimization, frontier molecular orbital (FMO) energies—specifically the energies of the highest occupied molecular orbital (HOMO) and lowest unoccupied molecular orbital (LUMO)—were taken from the output files of Gaussian calculation. These orbital energies were obtained from the self-consistent field (SCF) solution at the optimized geometry and are reported in unit of electron volts (eV).

The HOMO–LUMO energy gap (Δ*E*_H–L_) was calculated as:Δ*E*_H–L_ = *E*_LUMO_ – *E*_HOMO_*E*_LUMO_ and *E*_HOMO_ represent the orbital energies of the LUMO and HOMO, respectively. The *E*_HOMO_ reflects the molecule's ability to donate electrons, while the *E*_LUMO_ indicates its potential to accept electrons. The energy gap, Δ*E*_H–L_ provides crucial insights into the electronic excitation, chemical stability, reactivity, kinetic stability, and optical properties of the molecule.

The energy difference between the highest occupied molecular orbital (HOMO) and the lowest unoccupied molecular orbital (LUMO), commonly referred to as the HOMO–LUMO energy gap (Δ*E*_H–L_), is a fundamental electronic parameter that governs the chemical and physical behavior of molecular systems. A relatively small Δ*E*_H–L_ is generally indicative of enhanced chemical reactivity, reduced kinetic stability, and efficient intramolecular charge transfer, as electrons can be promoted more easily from the HOMO to the LUMO. In contrast, a larger HOMO–LUMO gap reflects increased molecular stability and diminished reactivity due to the higher energy required for electronic excitation. Consequently, the analysis of frontier molecular orbital energies provides valuable insight into the molecule's response in diverse chemical environments and is particularly important for evaluating its suitability for optoelectronic and photovoltaic applications.

Fig. 7 illustrates the spatial distribution of the HOMO and LUMO orbitals, along with the corresponding Δ*E*_H–L_, for complex 1. The HOMO is predominantly localized around the Ni(ii) metal center and the coordinated donor atoms, namely nitrogen and oxygen, indicating significant metal-ligand interaction in the electron-rich region of the molecule. In contrast, the LUMO is mainly delocalized over the ligand framework (HL), suggesting that electronic excitation involves charge transfer from the metal-centered region toward the ligand. The calculated HOMO and LUMO energies for complex 1 are −5.87 eV and −1.87 eV, respectively, resulting in a HOMO–LUMO energy gap of 4.00 eV. This moderate energy gap implies a balanced combination of stability and electronic responsiveness, highlighting the potential of complex 1 for applications requiring controlled charge-transfer processes.

### 4.2 NBO analysis

The computed bonding characteristics arising from interorbital interactions in the [Ni(L)_2_] (1) was investigated using NBO analysis. The NBO parameters represent the electronic wave functions described in terms of Lewis-type occupied orbitals and non-Lewis-type unoccupied localized orbitals. The secondary perturbation energies (*E*_i_) along with the corresponding donor–acceptor orbitals for the complex are presented in Table 3. As observed from Table 3, complex 1 possesses a higher *E*_i_ value, indicating stronger interorbital interactions. In case of the interaction of ligand (L1) with Ni(ii) ion, higher stabilization energy, *E*_i_ value of 44.38 kcal mol^−1^ and 36.12 kcal mol^−1^ correspond to the NBO charge transfer from LP (N_6_) to LP* (Ni) and LP (O_4_) to LP* (Ni) respectively. It signifies higher charge transfer from the counterpart L1 to Ni(ii) ion.^66^ Similarly, stronger interatomic orbital interaction was also observed for the interaction of ligand (L2) with Ni(ii) ion, having *E*_i_ value of 41.50 kcal mol^−1^ and 29.12 kcal mol^−1^ corresponding to the NBO charge transfer from LP (N_59_) to LP* (Ni) and LP (O_57_) to LP* (Ni) respectively.

**Table 3:** The secondary perturbation NBO energies (*E*_i_, in kcal mol^−1^) corresponding to the interatomic charge transfer interaction of the [Ni(L)_2_] (1) computed at the M06/def2svp level of theory

Interacting species	Interacting orbital		*E*_i_
	Donor/	Acceptor	
Ni to L1	LP* (Ni)	σ* (C_27_–H_28_)	4.36
	LP* (Ni)	σ* (O_4_–C_34_)	2.34
	LP* (Ni)	σ* (C_27_–H_29_)	2.44
	LP* (Ni)	σ* (N_6_–C_20_)	2.93
	LP (Ni)	σ* (N_6_–C_31_)	1.97
Ni to L2	LP (Ni)	σ* (N_59_–C_84_)	2.47
	LP* (Ni)	σ* (N_59_–C_73_)	4.10
	LP* (Ni)	σ* (O_57_–C_87_)	2.38
	LP* (Ni)	σ* (C_80_–H_82_)	4.35
L1 to Ni	LP (N_6_)	LP* (Ni)	44.38
	LP (O_4_)	LP* (Ni)	36.12
	*σ* (N_6_–C_31_)	LP* (Ni)	4.99
	*σ* (N_6_–C_20_)	LP* (Ni)	3.49
	*σ* (O_4_–C_34_)	LP* (Ni)	4.58
L2 to Ni	*σ* (O_57_–C_87_)	LP* (Ni)	4.40
	*σ* (O_59_–C_84_)	LP* (Ni)	5.09
	LP (O_57_)	LP* (Ni)	29.12
	LP (N_59_)	LP* (Ni)	41.50
L1 to L2	LP (O_4_)	LP (C_73_)	2.56
L2 to L1	LP (O_57_)	LP (C_20_)	2.99

## 5. Biomolecular interaction

### 5.1 DNA binding study

**Fig. 8:**
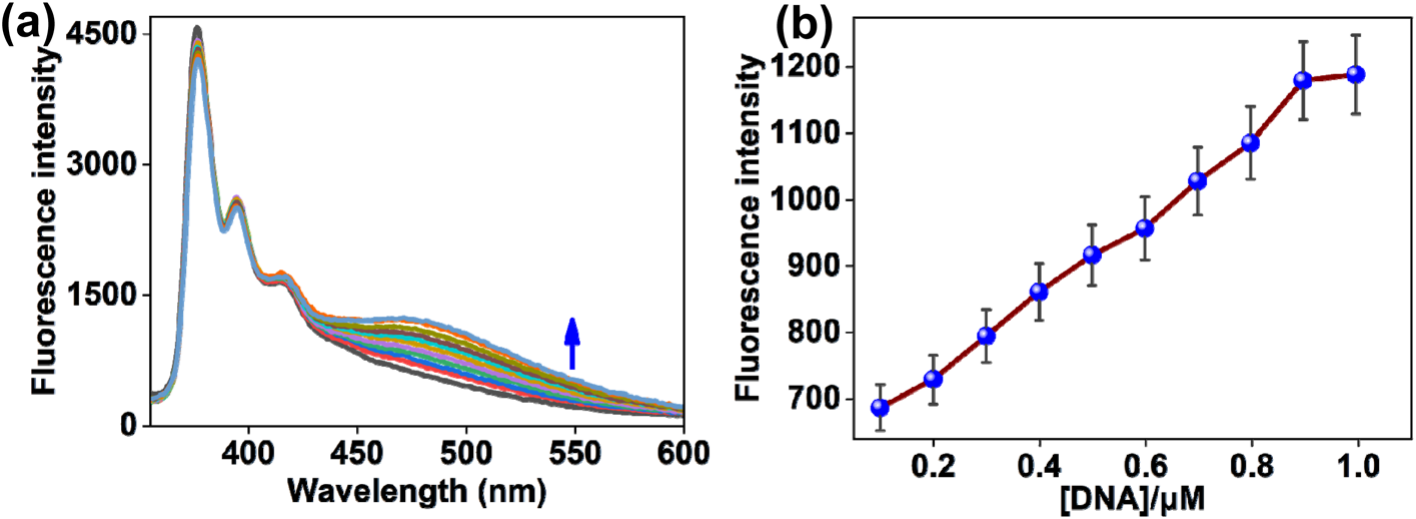
(a) Emission spectra of [Ni(L)_2_] (1) (*c* = 2.0 × 10^−5^ M) over serial increments of ct-DNA (*c* = 2 mM in base pairs) in a buffer medium of Tris-HCl (pH 7.2). (b) Changes emission intensity of the Ni(ii) complex 1 (*λ*_em_ = 475 nm) with respect to ct-DNA.

The DNA binding characteristics of [Ni(L)_2_] (1) with duplex ct-DNA was studied in Tris-HCl buffer at pH 7.2 by fluorescence spectrum analysis under the excitation wavelength at 335 nm. Initially, complex 1 exhibited emission signals at 376 nm, 394 nm, and 417 nm. But incremental additions of ct-DNA to solution of 1 led to a progressive increase of the red-shifted emission band at 475 nm (*λ*_ex_ = 335 nm) (Fig. 8). From the fluorescence studies, the detection limit of 1 towards ct-DNA was determined to be 0.075 µM (Fig. S6).

### 5.2 Protein binding study

**Fig. 9:**
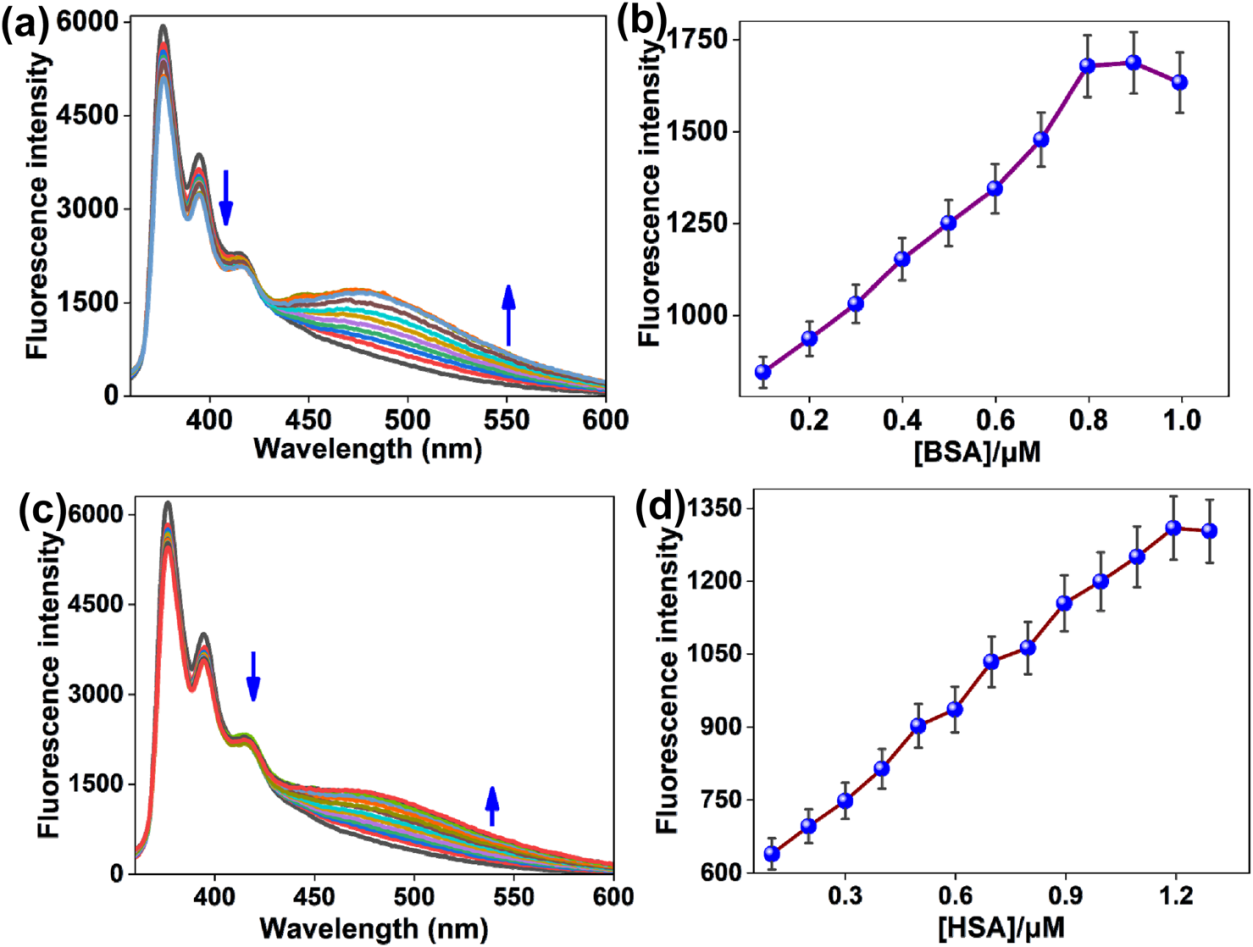
Emission spectra of [Ni(L)_2_] (1) (*c* = 2.0 × 10^−5^ M) over serial increments of (a) BSA (*c* = 7.4 µM) in a buffer medium of Tris-HCl buffer and (c) HSA (*c* = 7.4 µM) in a buffer medium of phosphate buffer (pH 7.2), respectively. Changes emission intensity of the Ni(ii) complex 1 (*λ*_em_ = 475 nm) with respect to (b) BSA and (d) HSA concentrations.

The binding capacity of [Ni(L)_2_] (1) with protein like BSA and HSA was investigated using fluorescence measurements in a Tris-HCl buffer at pH 7.2. With increasing concentration of BSA and HSA, emission intensity of the Ni(ii) complex 1 was decreased at 376 nm, 394 nm and 415 nm with the progressive enhancement of red-shifted emission band at 475 nm (*λ*_ex_ = 335 nm) (Fig. 9). From the fluorescence analysis, the detection limits for 1 against BSA and HSA were determined to be 0.188 µM and 0.122 µM, respectively (Fig. S7 and S8).

The appearance of a red-shifted emission band in the charged Ni-complex can be rationalized by changes in the electronic environment of the fluorophore upon interaction with surrounding charged or polar species. Coordination of the Ni with ligand HL increases the overall charge density and polarity of the complex, which stabilizes the excited state more effectively than the ground state. This stabilization reduces the energy gap between the excited and ground states (details in DFT study), resulting in a bathochromic (red) shift in the emission band. Additionally, electrostatic interactions between the charged nickel center and nearby biomolecules (such as DNA or proteins) can further restrict intramolecular motion of HL and suppress non-radiative decay pathways, leading to chelation-enhanced fluorescence (CHEF) accompanied by a red-shifted emission. Changes in solvent polarity and local dielectric constant around the charged Ni(ii) complex 1 upon binding also contribute to excited-state stabilization, reinforcing the observed red shifting of the fluorescence.^67,68^

## 6. *In silico* molecular docking studies

**Fig. 10:**
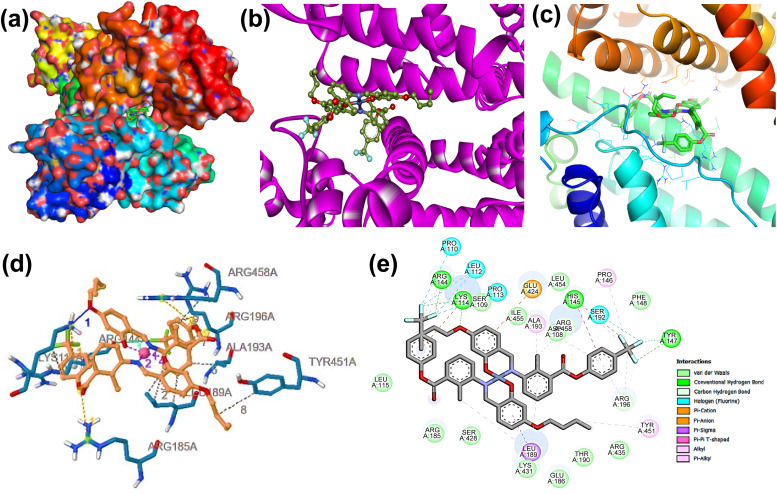
Possible interaction of complex [Ni(L)_2_] (1) at the binding site of 4JK4. (a) Three-dimensional surface (b and c) ribbon view (d) PLIP interactions, (e) two-dimensional interaction.

Molecular docking analysis was employed to further investigate the binding interactions of the complex [Ni(L)_2_] (1) with the BSA protein. This computational approach is commonly recognized as an efficient method for envisaging potential binding sites and affinities of metal complexes with biomolecules.^69,70^*In silico* molecular docking analysis disclosed that the docked complex fit well within the protein (BSA, PDB ID: 4JK4) binding site^71^]. Two and three-dimensional representations illustrating the best binding pose of the studied complex 1 with the target protein receptor (4JK4), corresponding to the highest total negative binding energy, are shown in Fig. 10. As illustrated in Fig. 10, multiple interactions comprising van der Waals forces, hydrophobic interactions, hydrogen bonding, halogen bonding interaction are observed between the amino acid active sites of the target protein, 4JK4 and the complex 1 supporting the inhibitory potential of this complex.^72^ The interactions resulted in a binding energy of −8.52 kcal mol^−1^ for the binding of 1 with the BSA receptor. This binding was stabilized primarily through interactions with amino acid residues, including ARG458A, ARG196A, ALA193A, TYR451A, ARG144A, LYS114A, and LEU189A. The hydrogen bond was between the LYS114A residue of 4JK4 and the NiL_2_ complex with a distance of 3.10 Å. The hydrophobic interaction forming residues are LEU189A, ALA193A, ARG196A, ARG458A, LYS114A, and TYR451A, while the halogen bond forming residue is ARG144A, and the π-cation interaction is with the residue, LYS114A of the target protein, 4JK4. The docking results indicate that the investigated complex 1 possesses strong and effective binding affinities. Overall, these docking results suggest that complex 1 exhibits significant binding affinity towards BSA, highlighting its strong biomolecular interaction potential.

## 7. Conclusion

In conclusion, we have designed, synthesized, and thoroughly characterized a new three-phenyl-ring-based bent-core Schiff base ligand (HL) and its mononuclear Ni(ii) complex [Ni(L)_2_] (1). The ligand (HL) exhibits aggregation-induced emission changes, with a red-shifted peak at 486 nm emerging beyond 60% water content. SEM analysis confirms distinct aggregation morphologies, correlating with the observed fluorescence behavior. The asymmetric unit of mononuclear Ni(ii) contains a Ni(ii) ion coordinated *via* imine nitrogen and phenoxido oxygen atoms of the deprotonated ligand (L), and a square planar geometry around the Ni(ii) center was observed. Topological analysis indicated that the underlying net has a point symbol of {336.448.57} and is of the bcu-x topological type. Computational analyses further underscore its structural stability and efficient charge transfer properties. Importantly, Ni(ii) complex 1 shows strong binding interactions with DNA, BSA, and HSA, evidenced by sensitive fluorescence responses. Its low detection limits (0.075–0.188 µM) highlight its high sensitivity for biomolecular recognition. Molecular docking results reveal that 1 bind strongly to BSA with a binding energy of −8.52 kcal mol^−1^, stabilized by multiple non-covalent interactions, which indicate the complex's strong biomolecular interaction potential, suggesting possible biological relevance.

## Conflicts of interest

No potential conflicts of interest was reported by the authors.

## Supplementary Material

RA-016-D5RA07894F-s001

RA-016-D5RA07894F-s002

## Data Availability

CCDC 2482583 contains the supplementary crystallographic data for this paper.^73^ The data supporting this article have been included as part of the supplementary information (SI). Supplementary information is available. See DOI: https://doi.org/10.1039/d5ra07894f.
