# Supplementary material for: Crystallographic and computational investigation of a bent-core Schiff base Ni(ii) complex with DNA and protein binding studies
Source: RSC Adv. 2026 Mar 5;16(14):12547–57. doi: 10.1039/d5ra07894f (PMC12961751; doi:10.1039/d5ra07894f)
Supplement: RA-016-D5RA07894F-s001 [file RA-016-D5RA07894F-s001.pdf]

Supplementary Information (SI) for RSC Advances.
This journal is © The Royal Society of Chemistry 2026

Crystallographic and Computational Investigation of a Bent-Core Schiff Base Ni(II) Complex with DNA and Protein Binding Studies

Kamrun Nahar Alia[a], Bugra Koknarugmani Debbarma[a], Sourav Nath[b,c], Subhadip Roy[d], Alan R. Kennedy[e], Suman Adhikari[c]*, Malavika S Kumar[f], Avijit Kumar Das*[f], Samiyara Begum[a]*, Golam Mohiuddin[a]*

[a]*Department of Chemistry, University of Science & Technology Meghalaya, Ri-Bhoi, Meghalaya 793101, India*

[b]*Department of Chemistry and Vivekananda Centre for Research, Ramakrishna Mission Residential College, Narendrapur, Kolkata-700103, India.*

[c]*Department of Chemistry, Govt. Degree College, Dharmanagar, Tripura(N)-799253, India.*

[d]*Department of Chemistry, The ICFAI University Tripura, Kamalghat, Mohanpur, Agartala, 799210, Tripura, India.*

[e]*Department of Pure and Applied Chemistry, University of Strathclyde, 295 Cathedral Street, Glasgow G1 1XL, Scotland, UK*

[f]*Department of Chemistry, Christ University, Hosur Road, Bangalore, Karnataka, 560029 India*
**Email: avijitkumar.das@christuniversity.in, golammohiuddin.ustm@gmail.com, Samiyara.ustm@gmail.com, sumanadhi@gmail.com*

Contents

1. Materials and physical measurements
2. X-ray crystallography and Topological analysis
3. Computational Details
4. Experimental for DNA and Protein binding studies
5. Molecular docking method

Figure S1. ^{1}H NMR spectra of bent-core Schiff base ligand **HL** in $CDCl_3$.
Figure S2. ^{13}C NMR spectra of bent-core Schiff base ligand **HL** in $CDCl_3$.
Figure S3. FT-IR spectrum of bent-core Schiff base ligand **HL**.
Figure S4. FT-IR spectrum of Ni(II) complex **1**.
Figure S5. HRMS spectrum of Ni(II) complex **1**.
Figure S6. ORTEP representation of the asymmetric unit of Ni(II) complex **1**, showing thermal ellipsoids drawn at the 50% probability level.
Figure S7. Overlay view of the X-ray (purple) and DFT structure (green), illustrating the close agreement between the experimental and theoretical geometries; only the major CF_3 component is shown.
Figure S8. Detection limit curve of DNA.
Figure S9. Detection limit curve of BSA.
Figure S10. Detection limit curve of HSA.
Figure S11. Comparative UV-visible spectra of Ni(II) complex **1.**
Figure S12. Comparative structure of Ni(II) complex **1.**
Figure S13. UV-vis spectra of **HL** ($c = 2\times10^{-5}$ M) in various organic solvents like CH_3CN, $CHCl_3$, C_2H_5OH, CH_3OH.
Figure S14. UV-vis spectra of **HL** and Ni(II) complex **1** ($c = 2\times10^{-5}$ M).
Table S1. Bond lengths for Ni(II) complex **1.**
Table S2. Bond angles for Ni(II) complex **1.**
Table S3. Cartesian coordinates of Ni(II) complex **1** optimized at M06/def2svp level.

1. Materials and physical measurements

All chemicals were reagent grade, obtained from commercial sources, and used without purification. IR spectra were recorded on an IR Prestige-21 Fourier Transform Infrared spectrophotometer Shimadzu (λ_{max} in cm^{-1}) on KBr disks. The nuclear magnetic resonance (NMR) spectra were recorded either on a JEOL AL-300 FTNMR or Bruker Avance III-400 spectrometer in $CDCl_3$ (chemical shift in δ) solution with TMS as internal standard. Mass spectra was carried out on a High-Resolution Mass Spectrometer (UHPLC-QTOF-HRMS), Make: Agilent, Model: G6546A. Elemental analysis was carried out in a Perkin Elmer 2500 series II elemental analyzer. UV-vis titration experiments were performed on a UV-Spectrophotometer: PerkinElmer, Lambda 30, and fluorescence experiment was done using Shimadzu RF-5301PC Fluorescence spectrofluorometer using a fluorescence cell of 10 mm path. SEM images were taken from a Field Emission Scanning Electron Microscope with EDAX, Thermoscientific, Model: Apreo S LoVac.

2. X-ray crystallography and Topological analysis

A yellow tablet-shaped-shaped crystal with dimensions 0.18×0.08×0.03 mm^3 was mounted. Data were collected using a Rigaku Synergy-i diffractometer operating at T = 100(2) K. Data were measured using omega scans with CuKα radiation. Data reduction, scaling, and absorption corrections were performed using CrysalisPro. The structure was solved by the ShelXT structure solution program [1] using iterative methods and refined by full matrix least squares minimisation on F^2 using a version of olex2.refine 1.5-dev [2]. All non-hydrogen atoms were refined anisotropically. Hydrogen atom positions were calculated geometrically and refined using the riding model. Bond distances and bond angles are listed in **Tables S1** and **S2**, respectively. The crystal data and structure refinement details are summarized in **Table 1**. In the complex, the $-CF_3$ group exhibits positional disorder over two sites, with a refined occupancy ratio of 0.623(5):0.377(5).

Topological analysis was carried out using the ToposPro software package in conjunction with the TTD database of periodic network topologies [3]. The network topologies were identified and described using the standard three-letter RCSR codes [4].

Topological analysis of coordination compounds enables the identification of correlations between the local coordination environment of building units (ligands and metal centers) and the overall network topology-defined by the connections among these centers[59]. This methodology supports the rational design of new structures and facilitates the exploration of relationships between topology and physical or chemical properties[60]. The local coordination

environment is often described using a coordination formula of the type $A^{n}D_{x}^{mbtq\cdots}$, where A represents the central atom, n is the number of coordinated bridging ligands, D indicates ligand denticity (e.g., mono-, bi-, tridentate), and m, b, t, q... denote the numbers of metal centers linked through mono-, bi-, tri-, and quadridentate coordination modes, respectively[61].

3. Computational Details

Geometry optimization:

The gas-phase geometry of the structure of Ni(II) complex $[Ni(L)_2]$ (**1**) was fully optimized by the Density functional of hybrid meta exchange-correlation functional (M06) along with the def2svp basis set. The vibrational frequency calculation was also carried out at the same level of theory to characterize that the obtained stationary point corresponds to a minimum on the potential energy surface having no imaginary frequency. Natural bond orbital (NBO) [5] analysis was performed at the same level of theory to examine various charge transfer interactions occurring between the interacting orbitals within the complex. All these computations were carried out using the Gaussian 09 program [6]. The optimized structure of $[Ni(L)_2]$ (**1**) was then utilized for molecular docking calculations.

4. Experimental for DNA and Protein binding studies

For UV-vis titrations of Ni(II) complex **1** with ct-DNA, BSA, and HSA, the stock solution of the Ni(II) complex (**1**) (c = 2× 10^{-5} M) was prepared in DMSO-Tris-HCl buffer (40 µL in 2 ml Tris-HCl buffer) at pH 7.2. And tris-HCl buffer was used to prepare the solution of ct-DNA (c = 2 mM in base pairs), BSA (c = 7.4 µM), and ovalbumin (c = 4.24 µM). The spectra of these solutions were recorded by means of UV-vis and fluorescence methods [7].

5. Molecular docking method

In-silico molecular docking serves as a highly valuable technique for understanding interactions between synthesized compounds and biological drug targets and thus plays a crucial role in drug discovery. In this study, the synthesized complex Ni(II) complex (**1**) was subjected to molecular docking with the protein receptor Bovine serum albumin (BSA) to elucidate drug–BSA interactions. We have assessed the preferred binding modes and energies of **1** at the receptor binding site. The docking was performed using AutoDock Tools (ADT) version 4.2.6 [8]. The crystal structure of BSA (PDB ID: 4JK4, resolution: 2.65Å) was selected as the receptor and prepared it for docking by removing all heteroatoms and water molecules, adding polar hydrogens, and Gasteiger charges. The binding sites were defined using a grid of interacting points. The default parameters of the free energy scoring function were applied to evaluate the binding affinity of **1** towards the receptor, 4JK4. The docked

conformations of the complex were further evaluated based on binding energy, hydrogen bonding, and hydrophobic interactions between the complex **1** and receptor, 4JK4.

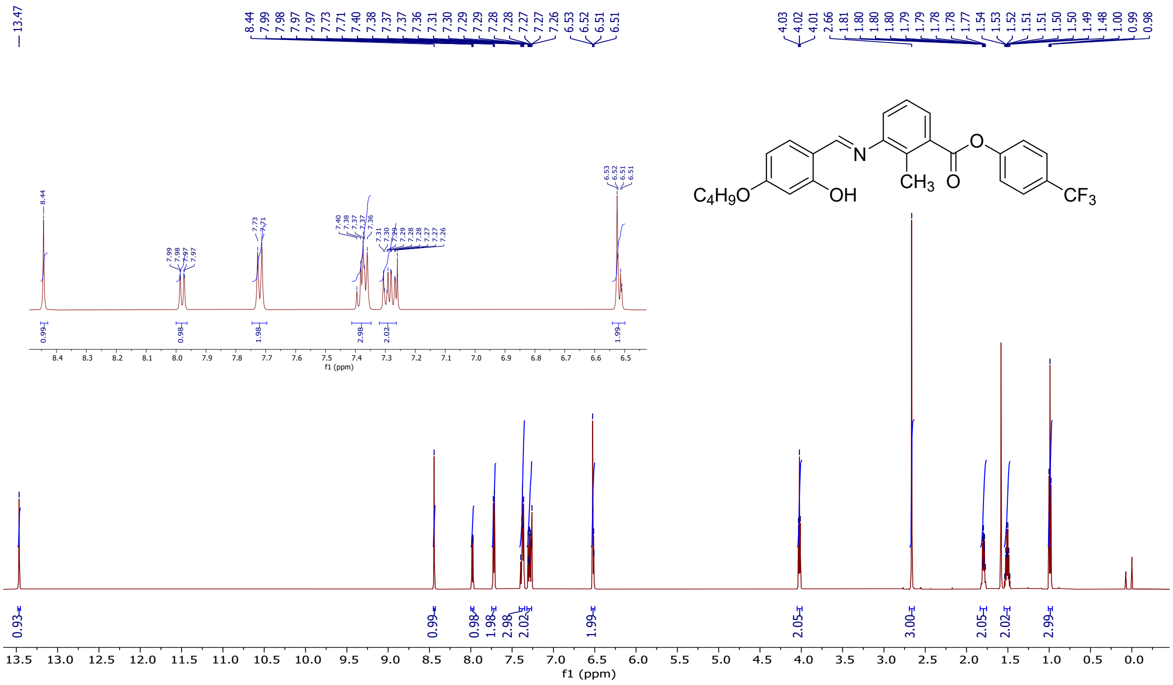

Figure S1. ^{1}H NMR spectra of bent-core Schiff base ligand **HL** in $CDCl_3$.

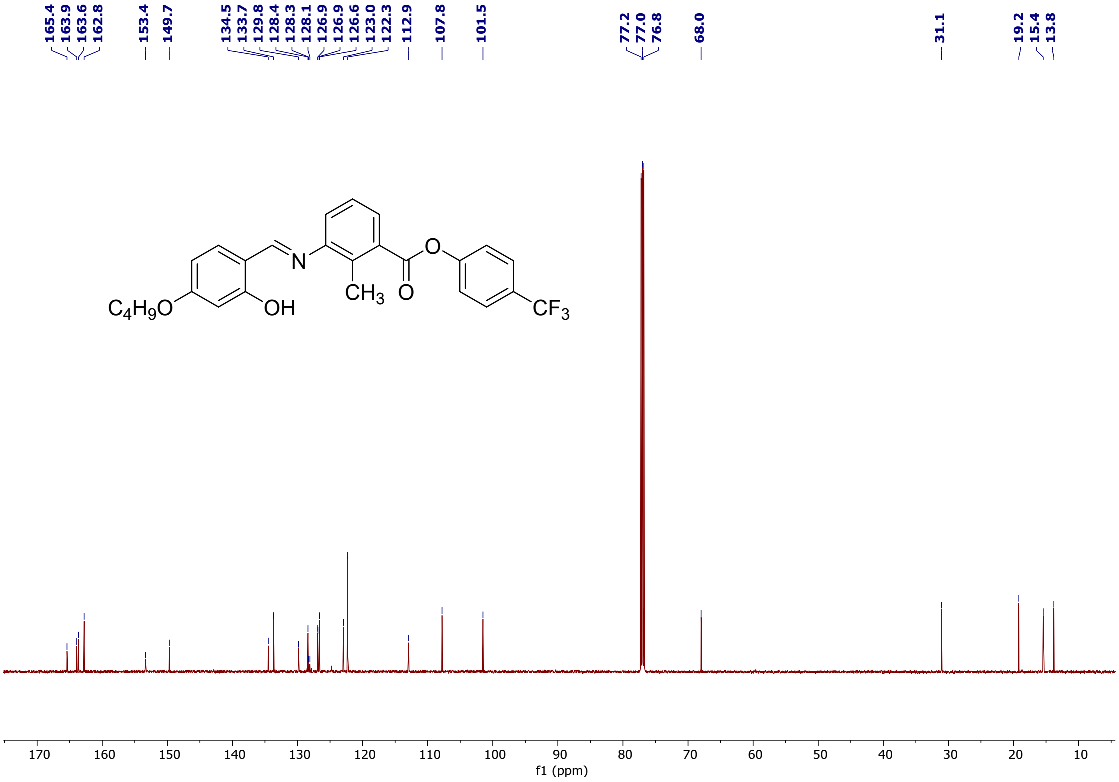

Figure S2. ^{13}C NMR spectra of bent-core Schiff base ligand **HL** in $CDCl_3$.

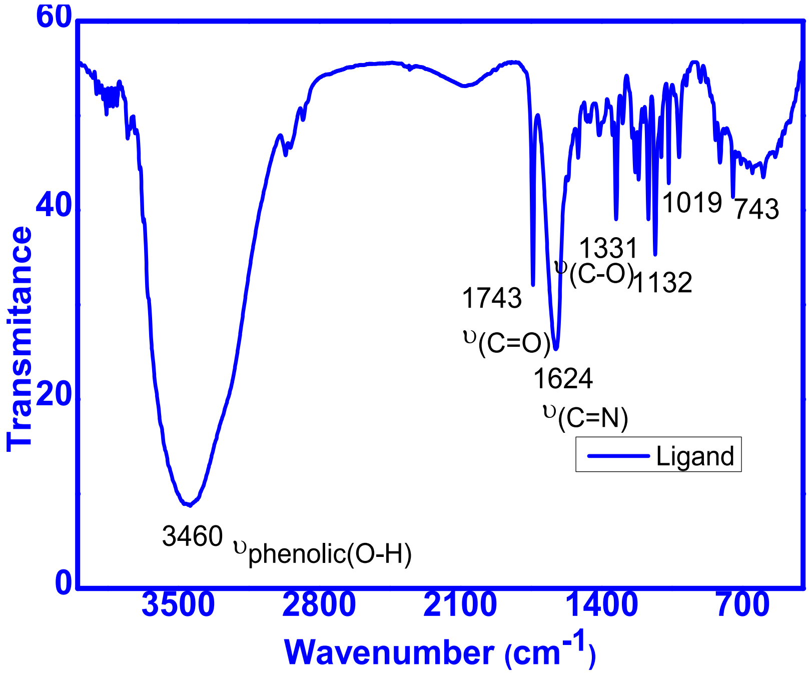

Figure S3. FT-IR spectrum of bent-core Schiff base ligand **HL**.

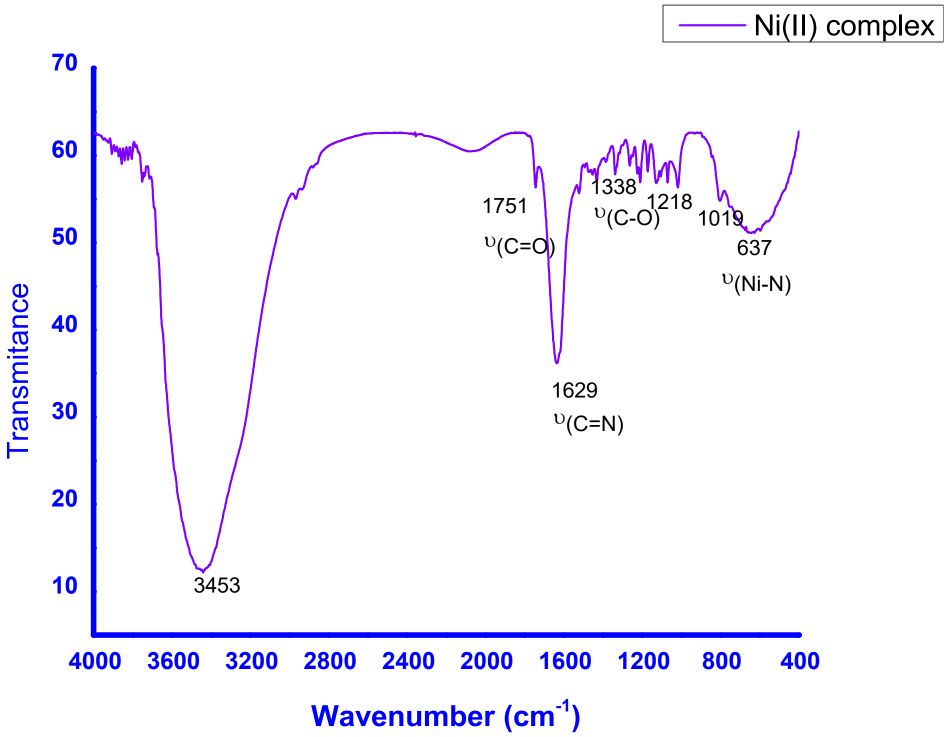

Figure S4. FT-IR spectrum of Ni(II) complex **1**.

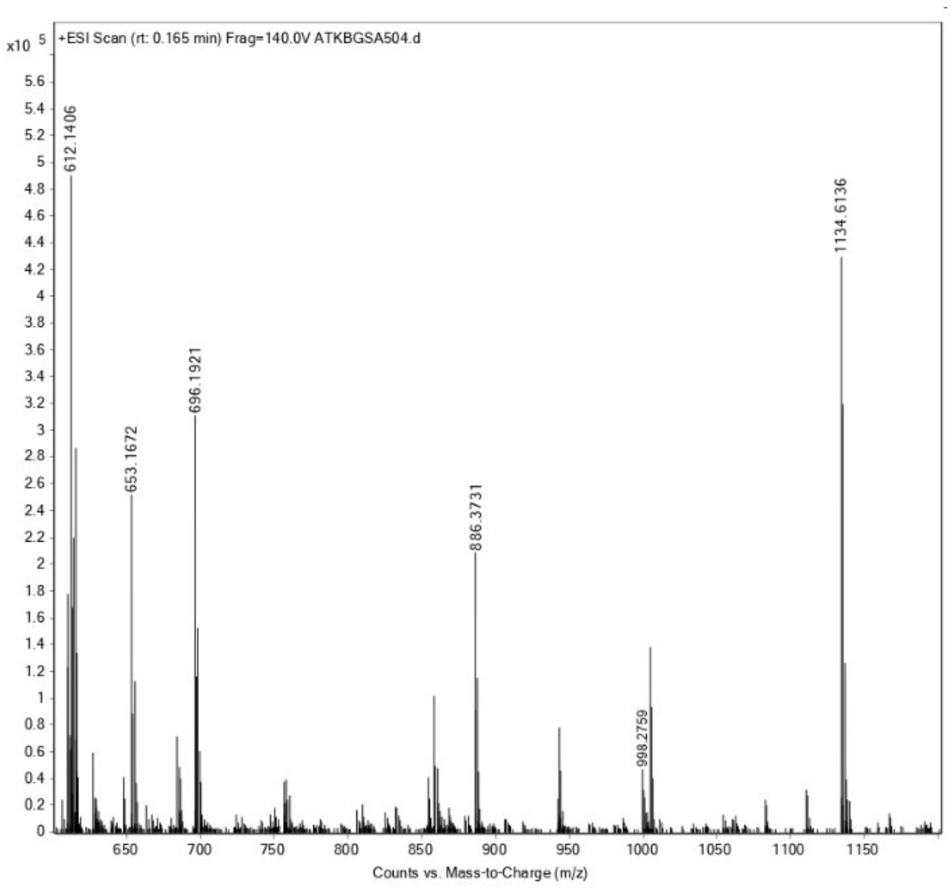

Figure S5. HRMS spectrum of Ni(II) complex **1** (water/methanol).

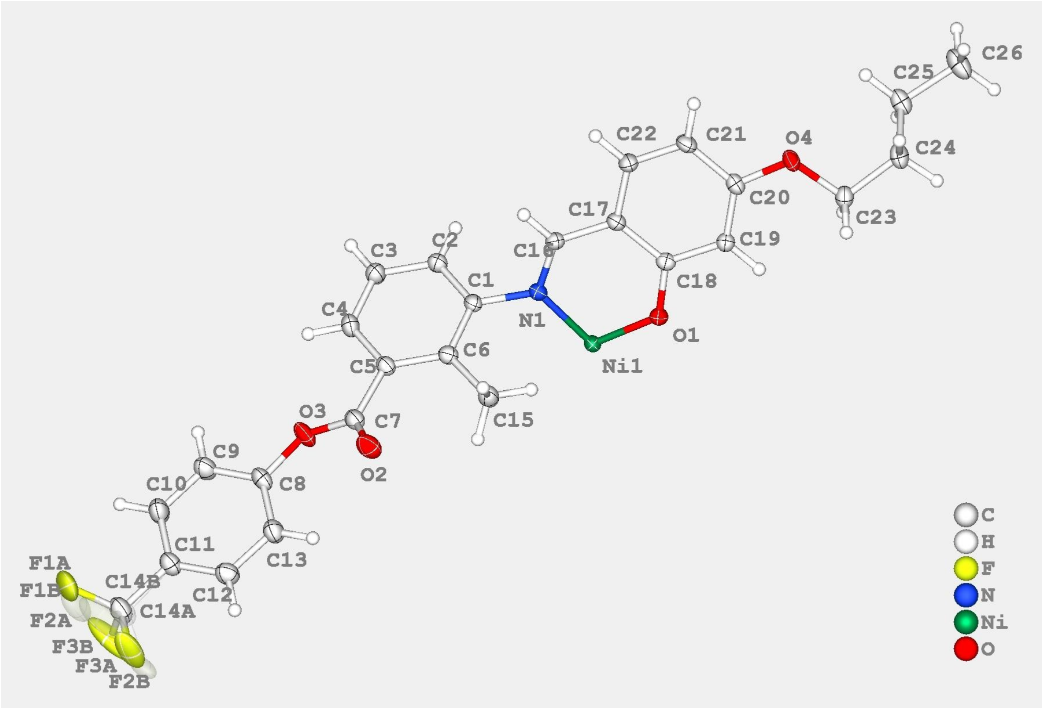

Figure S6. ORTEP representation of the asymmetric unit of Ni(II) complex **1**, showing thermal ellipsoids drawn at the 50% probability level.

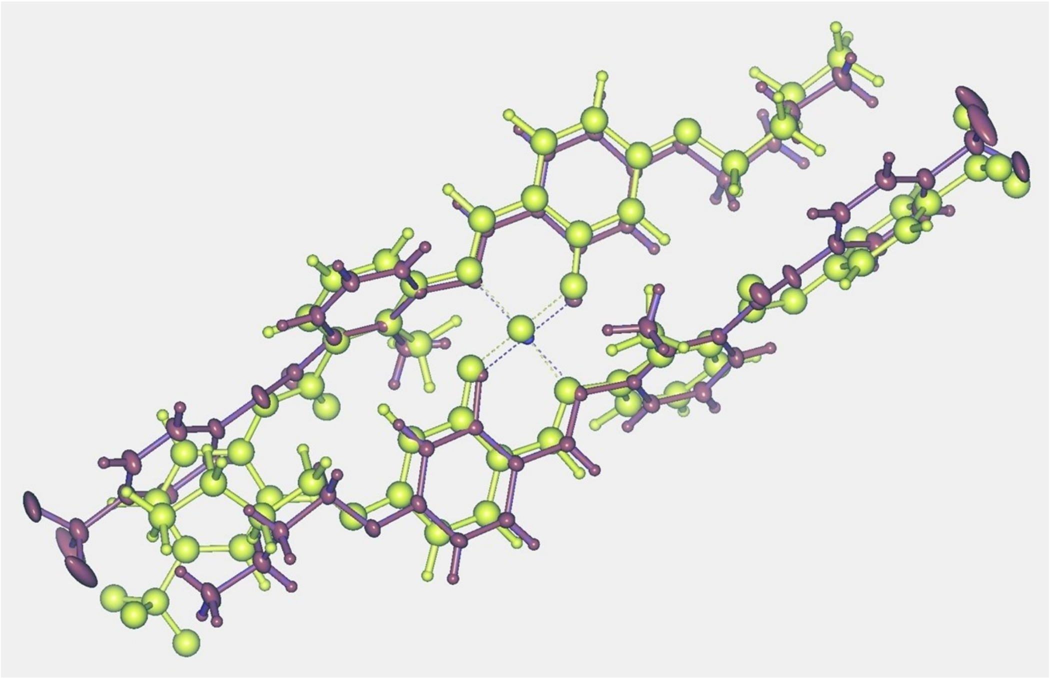

Figure S7. Overlay view of the X-ray (purple) and DFT structure (green), illustrating the close agreement between the experimental and theoretical geometries; only the major CF_3 component is shown.

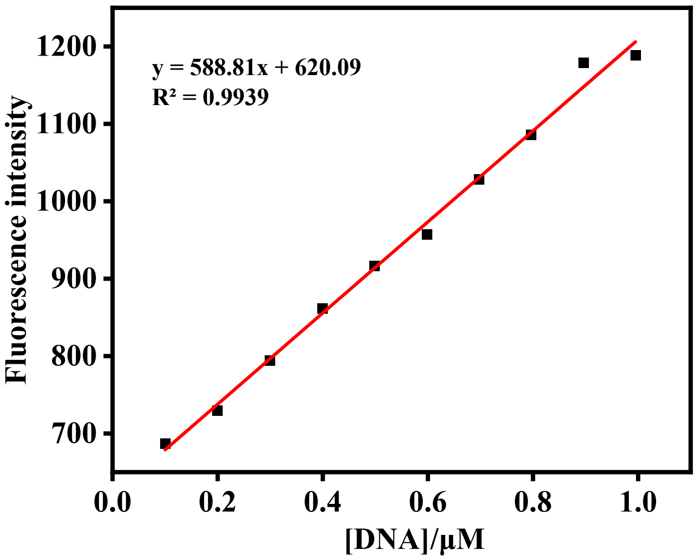

Figure S8. Detection limit curve of DNA.

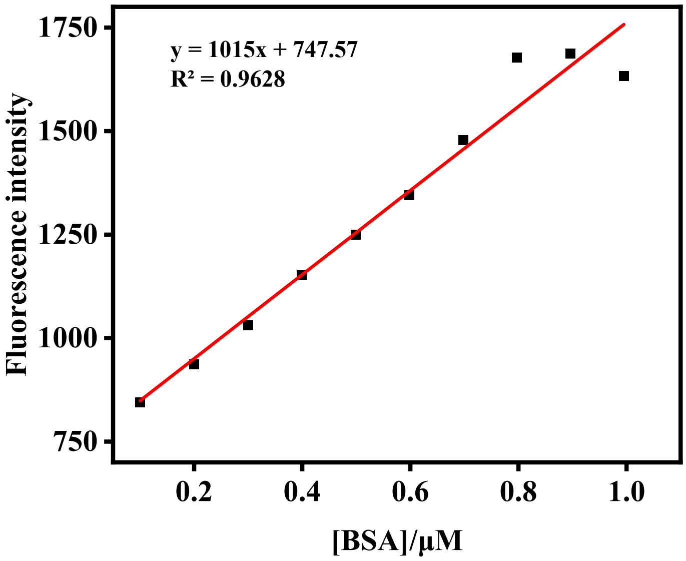

Figure S9. Detection limit curve of BSA.

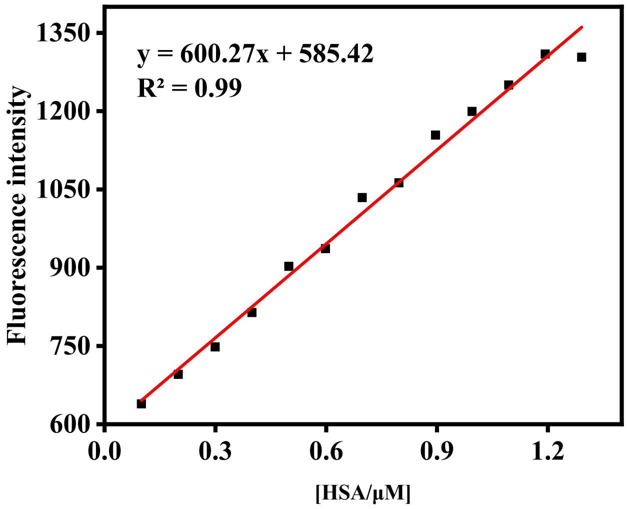

Figure S10. Detection limit curve of HAS.

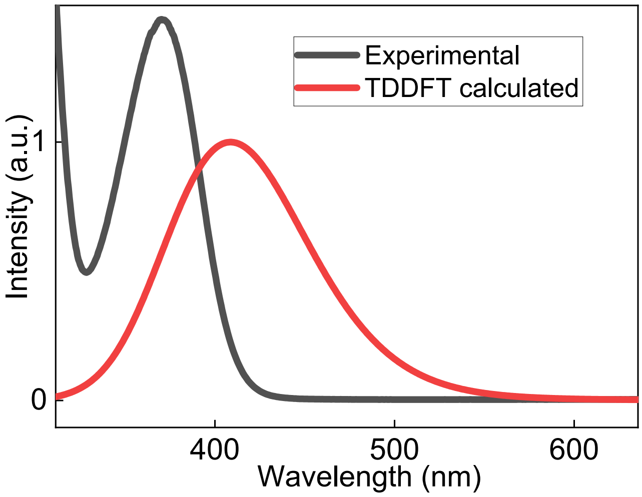

Figure S11. Comparative UV-visible spectra of Ni(II) complex **1.**

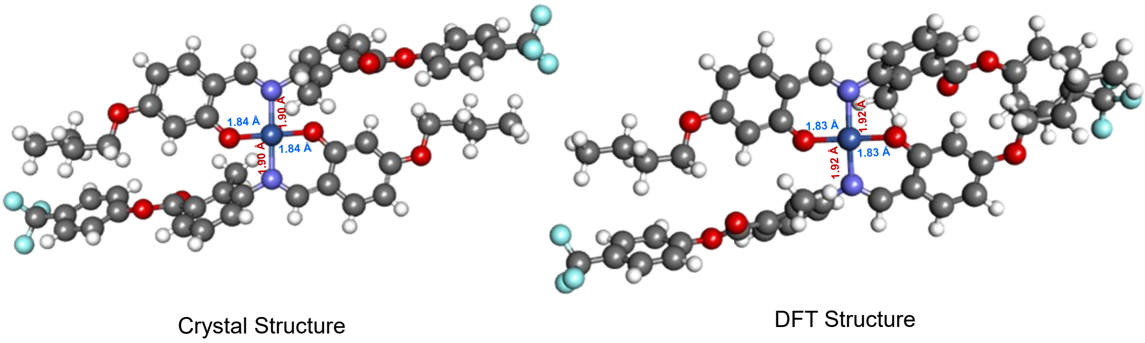

Figure S12. Comparative structure of Ni(II) complex **1.**

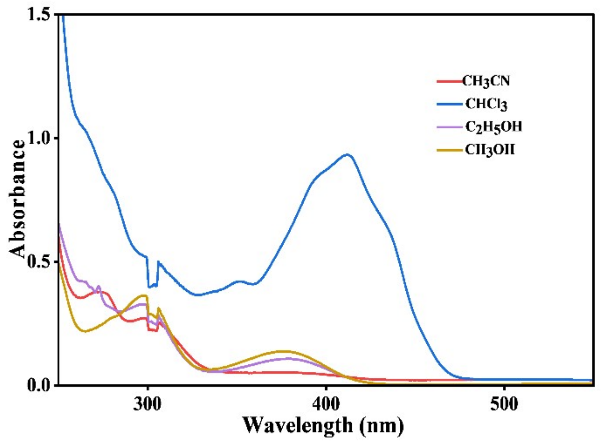

Figure S13. UV-vis spectra of **HL** (c = 2×10^{-5} M) in various organic solvents like CH_3CN, $CHCl_3$, C_2H_5OH, CH_3OH.

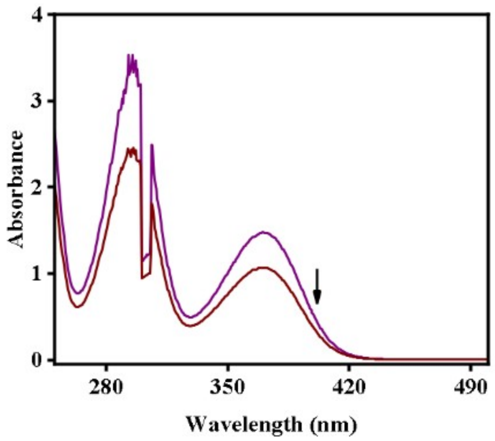

Figure S14. UV-vis spectra of **HL** and Ni(II) complex **1** (c = 2×10^{-5} M).

Table S1. Bond lengths for Ni(II) complex **1.**

Atom	Atom	Length/Å	Atom	Atom	Length/Å
Ni1	O1[1]	1.8341(14)	C5	C4	1.397(3)
Ni1	O1	1.8341(14)	C6	C1	1.408(3)
Ni1	N1	1.9033(17)	C6	C15	1.505(3)
Ni1	N1[1]	1.9033(17)	C1	C2	1.383(3)
O3	C8	1.394(2)	C2	C3	1.382(3)
O3	C7	1.374(3)	C3	C4	1.380(3)
O2	C7	1.196(3)	C16	C17	1.419(3)
O1	C18	1.312(2)	C17	C18	1.420(3)
O4	C20	1.360(2)	C17	C22	1.417(3)

Table S1. Bond lengths for Ni(II) complex **1.**

Atom	Atom	Length/Å	Atom	Atom	Length/Å
O4	C23	1.439(2)	C18	C19	1.411(3)
N1	C1	1.444(3)	C19	C20	1.385(3)
N1	C16	1.308(3)	C20	C21	1.409(3)
C11	C12	1.382(3)	C21	C22	1.368(3)
C11	C10	1.386(3)	C23	C24	1.515(3)
C11	C14A	1.497(5)	C24	C25	1.523(3)
C11	C14B	1.468(7)	C25	C26	1.518(3)
C12	C13	1.387(3)	C14A	F1A	1.324(6)
C13	C8	1.381(3)	C14A	F2A	1.308(6)
C8	C9	1.385(3)	C14A	F3A	1.306(6)
C9	C10	1.385(3)	C14B	F1B	1.313(8)
C7	C5	1.492(3)	C14B	F2B	1.317(8)
C5	C6	1.403(3)	C14B	F3B	1.295(8)

[1]1-X,1-Y,1-Z

Table S2. Bond angles for Ni(II) complex **1.**

Atom	Atom	Atom	Angle/°	Atom	Atom	Atom	Angle/°
O1	Ni1	O1[1]	180.0	C6	C1	N1	119.78(18)
N1[1]	Ni1	O1[1]	92.73(6)	C2	C1	N1	118.43(17)
N1[1]	Ni1	O1	87.27(6)	C2	C1	C6	121.74(18)
N1	Ni1	O1[1]	87.27(6)	C3	C2	C1	120.20(19)
N1	Ni1	O1	92.73(6)	C4	C3	C2	119.6(2)
N1[1]	Ni1	N1	180.0	C3	C4	C5	120.49(19)
C7	O3	C8	124.71(17)	C17	C16	N1	125.53(19)
C18	O1	Ni1[1]	126.21(13)	C18	C17	C16	121.32(18)
C23	O4	C20	118.82(16)	C22	C17	C16	119.83(18)
C1	N1	Ni1	119.80(13)	C22	C17	C18	118.74(18)
C16	N1	Ni1	123.12(14)	C17	C18	O1	122.80(18)
C16	N1	C1	116.40(17)	C19	C18	O1	117.92(18)
C10	C11	C12	120.1(2)	C19	C18	C17	119.28(18)
C14A	C11	C12	120.7(3)	C20	C19	C18	119.79(19)
C14A	C11	C10	119.2(3)	C19	C20	O4	124.06(19)
C14B	C11	C12	119.4(4)	C21	C20	O4	114.45(18)
C14B	C11	C10	120.2(4)	C21	C20	C19	121.49(18)
C14B	C11	C14A	4.6(5)	C22	C21	C20	118.82(19)
C13	C12	C11	120.7(2)	C21	C22	C17	121.83(19)
C8	C13	C12	118.7(2)	C24	C23	O4	107.04(17)
C13	C8	O3	125.4(2)	C25	C24	C23	113.27(18)
C9	C8	O3	113.05(19)	C26	C25	C24	113.2(2)
C9	C8	C13	121.3(2)	F1A	C14A	C11	113.6(4)

Table S2. Bond angles for Ni(II) complex **1.**

Atom	Atom	Atom	Angle/°	Atom	Atom	Atom	Angle/°
C10	C9	C8	119.5(2)	F2A	C14A	C11	111.0(4)
C9	C10	C11	119.7(2)	F2A	C14A	F1A	103.8(5)
O2	C7	O3	123.42(19)	F3A	C14A	C11	114.6(4)
C5	C7	O3	109.01(18)	F3A	C14A	F1A	103.2(4)
C5	C7	O2	127.57(19)	F3A	C14A	F2A	109.9(5)
C6	C5	C7	121.53(19)	F1B	C14B	C11	115.5(6)
C4	C5	C7	117.51(18)	F2B	C14B	C11	115.3(6)
C4	C5	C6	120.95(19)	F2B	C14B	F1B	104.2(7)
C1	C6	C5	116.92(19)	F3B	C14B	C11	111.7(6)
C15	C6	C5	123.08(18)	F3B	C14B	F1B	104.9(8)
C15	C6	C1	119.99(18)	F3B	C14B	F2B	104.1(7)

[1]1-X,1-Y,1-Z

Table S3. Cartesian coordinates of Ni(II) complex **1** optimized at M06/def2svp level.

Center Number	Atomic Number	Atomic Type	Coordinates (Angstroms) X	Y	Z
1	28	0	0.046456	0.246575	0.198205
2	8	0	6.543713	1.357546	0.272163
3	8	0	5.231652	1.242138	-1.548696
4	8	0	-1.570550	0.880411	-0.407141
5	8	0	-5.745236	3.012614	0.215710
6	7	0	0.579599	1.992804	0.804742
7	6	0	9.863491	-0.704881	-1.085629
8	6	0	8.596371	-1.187625	-1.403594
9	1	0	8.493731	-2.123700	-1.957610
10	6	0	7.456790	-0.497693	-1.000644
11	1	0	6.463581	-0.889305	-1.229743
12	6	0	7.604089	0.687079	-0.280937
13	6	0	8.869773	1.189106	0.023293

14	1	0	8.943512	2.121374	0.588962
15	6	0	9.999011	0.489511	-0.374785
16	1	0	10.996351	0.867016	-0.128920
17	6	0	5.346333	1.492978	-0.381496
18	6	0	4.282802	1.936965	0.552351
19	6	0	2.932936	1.911255	0.140076
20	6	0	1.951162	2.178774	1.118316
21	6	0	2.308121	2.538387	2.415588
22	1	0	1.516358	2.709641	3.151381
23	6	0	3.646787	2.625070	2.786601
24	1	0	3.917472	2.910430	3.806411
25	6	0	4.627908	2.301954	1.865312
26	1	0	5.680055	2.317852	2.154646
27	6	0	2.513445	1.572585	-1.255720
28	1	0	3.075821	2.155817	-1.997529
29	1	0	1.439439	1.750937	-1.401949
30	1	0	2.727734	0.514787	-1.490884
31	6	0	-0.246222	2.981899	1.003718
32	1	0	0.174613	3.924451	1.397004
33	6	0	-1.639306	2.971811	0.750395
34	6	0	-2.242187	1.876960	0.055067
35	6	0	-3.643917	1.902305	-0.145739
36	1	0	-4.078462	1.049622	-0.671681
37	6	0	-4.411667	2.961346	0.315001
38	6	0	-3.801457	4.076702	0.947412
39	1	0	-4.438984	4.900229	1.275877
40	6	0	-2.446650	4.066712	1.149664

41	1	0	-1.964658	4.912311	1.653144
42	6	0	-6.465995	1.847709	-0.150885
43	1	0	-6.176982	1.009563	0.513425
44	1	0	-6.205860	1.546555	-1.189147
45	6	0	-7.938133	2.150300	-0.046566
46	1	0	-8.150064	2.519617	0.974232
47	1	0	-8.492320	1.198782	-0.149537
48	6	0	-8.436771	3.147250	-1.077911
49	1	0	-7.856633	4.083215	-0.987880
50	1	0	-8.213706	2.755271	-2.090283
51	6	0	-9.920272	3.424661	-0.942517
52	1	0	-10.509570	2.496167	-1.034165
53	1	0	-10.283666	4.129777	-1.706054
54	1	0	-10.155213	3.856916	0.044825
55	8	0	-6.299411	-1.484446	-1.720825
56	8	0	-5.517272	-1.468101	0.389832
57	8	0	1.573352	-0.415032	0.994282
58	8	0	5.750514	-2.640372	1.165998
59	7	0	-0.337364	-1.441289	-0.654595
60	6	0	-10.350355	-0.946765	-1.023689
61	6	0	-9.607918	-1.685455	-0.106585
62	1	0	-10.102849	-2.126049	0.762053
63	6	0	-8.240118	-1.876870	-0.287996
64	1	0	-7.662305	-2.454993	0.432010
65	6	0	-7.622201	-1.314319	-1.404405
66	6	0	-8.361181	-0.571003	-2.329041
67	1	0	-7.844059	-0.148821	-3.194868

68	6	0	-9.722479	-0.389316	-2.140298
69	1	0	-10.306972	0.187408	-2.864334
70	6	0	-5.299306	-1.544141	-0.790194
71	6	0	-3.972933	-1.652501	-1.443625
72	6	0	-2.792945	-1.619314	-0.663746
73	6	0	-1.564482	-1.599411	-1.355720
74	6	0	-1.520503	-1.664658	-2.747120
75	1	0	-0.546530	-1.615085	-3.244083
76	6	0	-2.688749	-1.751397	-3.496754
77	1	0	-2.642059	-1.807384	-4.587170
78	6	0	-3.908735	-1.727335	-2.846233
79	1	0	-4.835548	-1.759251	-3.419640
80	6	0	-2.797762	-1.594787	0.830901
81	1	0	-3.399584	-2.417472	1.244095
82	1	0	-1.777016	-1.664272	1.228622
83	1	0	-3.257781	-0.668612	1.212594
84	6	0	0.569769	-2.363561	-0.831576
85	1	0	0.289150	-3.230837	-1.455453
86	6	0	1.884127	-2.387778	-0.304212
87	6	0	2.322252	-1.387927	0.618733
88	6	0	3.638614	-1.479564	1.140155
89	1	0	3.934562	-0.703568	1.850743
90	6	0	4.485318	-2.506407	0.746832
91	6	0	4.044868	-3.502775	-0.164752
92	1	0	4.741588	-4.297811	-0.439005
93	6	0	2.770189	-3.435375	-0.661209
94	1	0	2.416355	-4.201780	-1.359916

95	6	0	6.299441	-1.690188	2.057467
96	1	0	6.158155	-0.667425	1.652722
97	1	0	5.763044	-1.727053	3.027729
98	6	0	7.767881	-1.986259	2.222794
99	1	0	7.895046	-2.992092	2.663957
100	1	0	8.223098	-2.034566	1.215783
101	6	0	8.489185	-0.941463	3.055587
102	1	0	8.073968	-0.926523	4.081449
103	1	0	8.277160	0.061985	2.634680
104	6	0	9.986977	-1.174348	3.096744
105	1	0	10.417677	-1.161982	2.079676
106	1	0	10.510143	-0.410840	3.693062
107	1	0	10.225684	-2.158073	3.534961
108	6	0	-11.826275	-0.744501	-0.857159
109	9	0	-12.496387	-1.191334	-1.921469
110	9	0	-12.131805	0.551565	-0.732428
111	9	0	-12.305562	-1.370860	0.213659
112	6	0	11.104168	-1.468200	-1.433776
113	9	0	12.000768	-0.694668	-2.048068
114	9	0	10.856626	-2.507732	-2.225383
115	9	0	11.699316	-1.938979	-0.330170

References:

1. G. M. Sheldrick, Acta Cryst., 2015, C71, 3-8

2. L.J. Bourhis, O.V. Dolomanov, R.J. Gildea, J.A.K. Howard, H. Puschmann, Acta Crystallographica Section A, 71 (2015) 59-75. Doi: **10.1107/S2053273314022207**

3. V.A. Blatov, A.P. Shevchenko, D.M. Proserpio, Cryst. Growth Des., 14 (2014) 3576-3586. Doi: 10.1021/cg500498k

4. M. O'Keeffe, M.A. Peskov, S.J. Ramsden, O.M. Yaghi, Acc. Chem. Res., 41 (2008) 1782-1789. Doi: 10.1021/ar800124u

5. Reed, A. E.; Weinhold, F.; Curtiss, L. A.; Pochatko, D. J. Natural bond orbital analysis of molecular interactions: theoretical studies of binary complexes of HF, H_2O, NH_3, N_2, O_2, F_2, CO and CO_2 with HF, H_2O and NH_3. J. Chem. Phys. 1986, 84, 5687−5705.

6. Frisch MJ, Trucks GW, Schlegel HB, Scuseria GE, Robb MA, Cheeseman JR, *et al*. Gaussian 09, Revision A.02. Wallingford CT: Gaussian, Inc.; 2016.

7. Gurusamy, S., Krishnaveni, K., Sankarganesh, M., Asha, R. N., & Mathavan, A. (2022). Synthesis, characterization, DNA interaction, BSA/HSA binding activities of VO (IV), Cu (II) and Zn (II) Schiff base complexes and its molecular docking with biomolecules. *Journal of Molecular Liquids*, *345*, 117045.

8. Morris, G. M.; Huey, R.; Lindstrom, W.; Sanner, M. F.; Belew, R. K.; Goodsell, D. S.; Olson, A. J. AutoDock4 and AutoDockTools4: Automated docking with selective receptor flexibility. *J. Comput. Chem.* **2009**, *30* (16), 2785–2791. https://doi.org/10.1002/jcc.21256.
